# Supplementary material for: Attachment and antibiotic response of early-stage biofilms studied using resonant hyperspectral imaging
Source: NPJ Biofilms Microbiomes. 2020 Nov 27;6:57. doi: 10.1038/s41522-020-00169-1 (PMC7695833; doi:10.1038/s41522-020-00169-1)
Supplement: Supplementary file 1 — Supplementary Information [file 41522_2020_169_MOESM1_ESM.pdf]

Attachment and antibiotic response of early-stage biofilms studied using resonant hyperspectral imaging

Yue Wang[1], Christopher P. Reardon[1], Nicholas Read[2], Stephen Thorpe[2], Adrian Evans[3], Neil Todd[3], Marjan Van Der Woude[4] and Thomas F. Krauss[1]

1. Department of Physics, University of York, Heslington, York, North Yorkshire, YO10 5DD, UK
2. Department of Biology, University of York, Heslington, York, North Yorkshire, YO10 5DD, UK
3. York Teaching Hospital NHS Foundation Trust, The York Hospital, York, North Yorkshire, YO31 8HE, UK
4. York Biomedical Research Institute, Hull York Medical School University of York, Heslington, York, North Yorkshire, YO10 5DD, UK

Corresponding author: Dr Yue Wang, Department of Physics, University of York, York YO10 5DD UK, tel: +44 1904322700, email: yue.wang@york.ac.uk

Supplementary information

I. Hyperspectral image vs. confocal microscope image

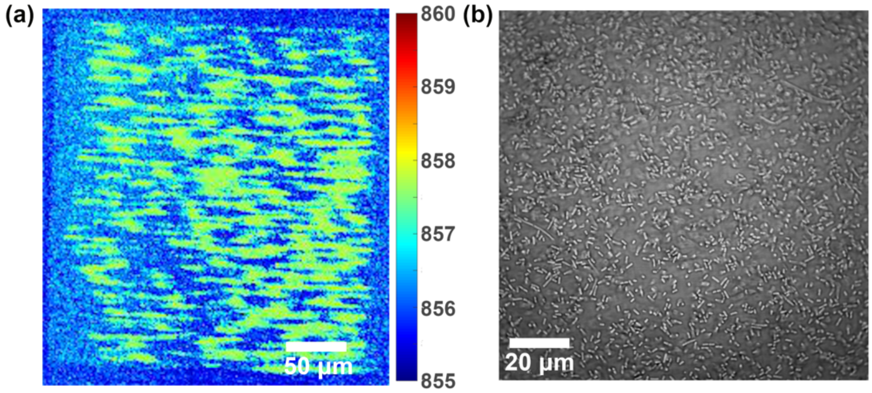

Supplementary Figure 1 (a) Hyperspectral image at 5h from the start of the experiment with initial e.coli concentration of $2x10^8$ CFU per mL; (b) a corresponding confocal microscope image of the same biofilm.

II. Uncertainty in resonance wavelength

The uncertainty in resonance wavelength, λ_n, is determined by monitoring the grating's resonance with a constant water flow. Over 7.5 h, the standard deviation (σ) of the resonance wavelength is measured to be 0.037 nm. And we estimate the smallest detectable shift to be 3σ, which is 0.111 nm. The resonance wavelength shift, which is defined as $\Delta\lambda = \lambda_n - \lambda_0$, has a standard deviation σ of 0.055 nm and 3σ of 0.157 nm.

III. Baclight Live/Dead viability assays

Samples are prepared in the flow cell for various lengths of time, i.e. 1 h, 3.5 h, 5 h and 10 h, with initial *E.coli* TG1 concentration of 2 x 10^8 CFU per mL. The gratings are then taken out from the flow cell, gently washed three times with phosphate buffered saline (PBS) buffer three times and stained with the Baclight Live/Dead viability assay. The washing steps are essential for removing loosely attached non-biofilm cells. The samples are then incubated at room temperature in the dark for 15 minutes. Fluorescent images are then taken with a ZEISS LSM 880 single-photon confocal microscope. It is worth noting here that for the short-time-grown sample (1 h in the flow cell), some bacteria were washed away from the grating surface during the staining process, due to weak attachment to the surface during this stage - this image therefore does not reflect the true nature of the sample in this case.

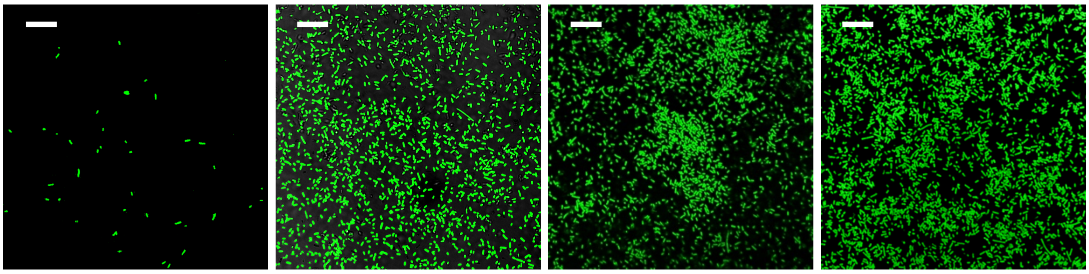

Supplementary Figure 2. Confocal microscopic images of early-stage biofilms on the sensor surface. 1 h, 3.5 h, 5 h and 10 h biofilm samples are stained with the Live/Dead BacLight viability assay and examined with a ZEISS LSM 800 confocal microscope. The scale bars in the micrographs are all 10 μm long.

IV. Biofilm formation time

The relationship between the bacterial seeding density and the "planktonic" phase time t_{plank} is plotted in **Supplementary Figure 3**. Although it is intuitive that a lower initial inoculum

density leads to a longer biofilm formation time, the technique readily allows us to quantify this formation time.

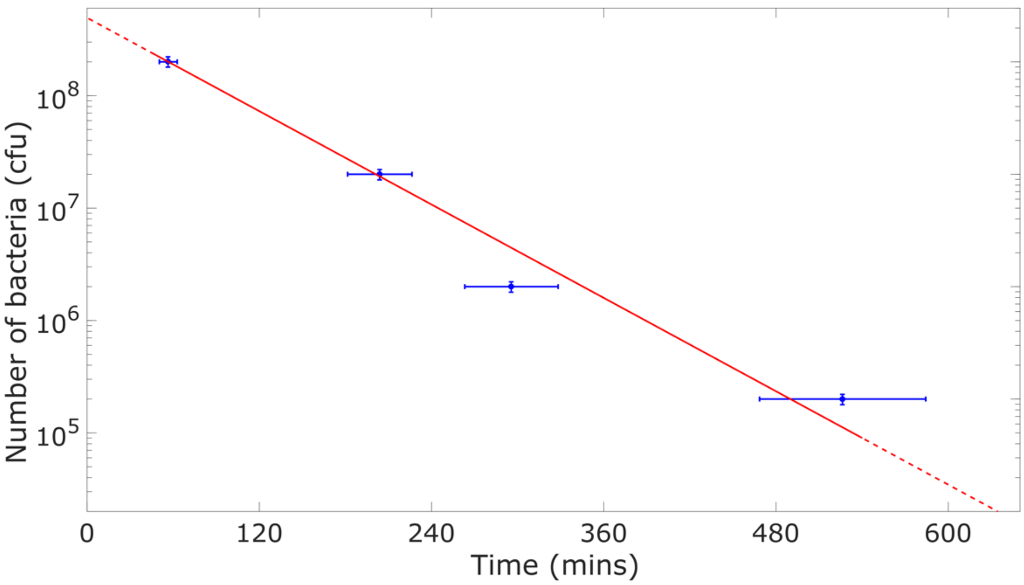

Supplementary Figure 3. Estimation of initial bacterial numbers. Relationship between the initial bacterial concentration and the "planktonic" phase time duration of the early-stage biofilm (t_{plank} in Eq.1) in a log-linear plot. The solid line is a linear fit to the data points. The error bars represent the standard deviations of repeated experiments.

V. Determining the MICs

We use a resazurin-based microdilution assay to determine the minimum inhibitory concentrations (MICs) of different drugs. Trimethoprim, nitrofurantoin, rifampicin and ciprofloxacin are diluted from individual stock solutions to 1024 μg mL^{-1}. In a 96-well plate, column 1 of is used as a no antibiotic control and column 12 contains only LB broth as a sterility control (see Supplementary Figure 4.). For the antibiotic serial dilution, 50 μL of the 1024 μg mL^{-1} antibiotic solution is first added to the 512 μg mL^{-1} column, and is well mixed with the liquid culture using a multichannel pipette before transferring to the next well, and repeated twice, to give a range of concentrations between 1 to 512 μg mL^{-1}, see Supplementary Figure 4.

E. coli TG1 are grown overnight in LB broth at 37ºC. Cells are washed three times in Mueller Hinton broth (MH) then re-suspended in MH before first being diluted to 0.1 OD to give

standardised suspension of 10^8 CFU per mL. The standardised suspension is diluted 1:100 then 50 µL added to each well to give a final bacteria concentration of 5.5×10^5 CFU per mL. 50 µL MHB is added to column 12 to give 100 µL of solution in all wells. After overnight incubation at 37°C, 30 µL of 0.015% solution of resazurin in MHB is introduced in each well and incubated for 4 hours. Results are validated by visual inspection of the wells.

The MIC microdilution experiments have been repeated for four times (twice with resazurin dye, twice without dye but the growth curves were monitored). The MICs of each drug are 0-1 µg mL^{-1} (trimethoprim), 4-8 µg mL^{-1} (nitrofurantoin), 8-16 µg mL^{-1} (rifampicin) and 0-1 µg mL^{-1} (ciprofloxacin). The MIC measurements were all performed with OD = 0.2, i.e. 2 x 10^8 CFU per mL diluted from overnight cultures.

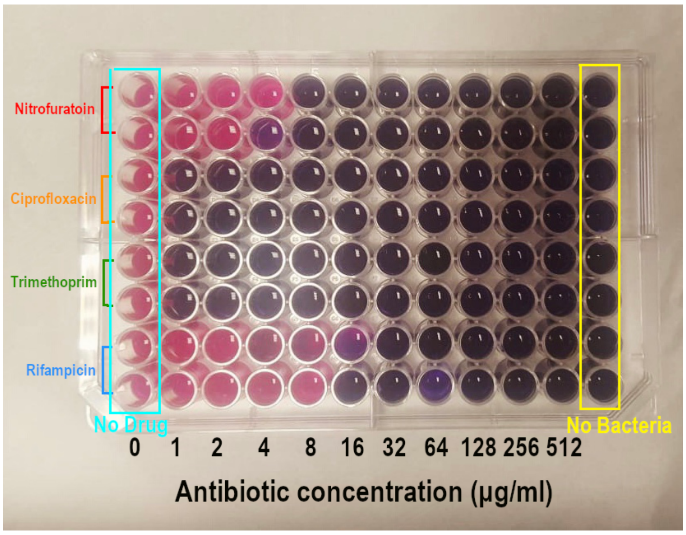

Supplementary Figure 4. A resazurin microdilution assay with four different antibiotics. The concentration of the four antibiotics, nitrofurantoin, ciprofloxacin, trimethoprim and rifampicin, is increased along the columns from 0 to 512 µg mL^{-1}. Each antibiotic has been tested in duplicate. The first column has no antibiotic and the last column has only LB broth with no bacteria. Pink colour indicates growth and dark purple means inhibition of growth.

VI. Antibiotics susceptibility test

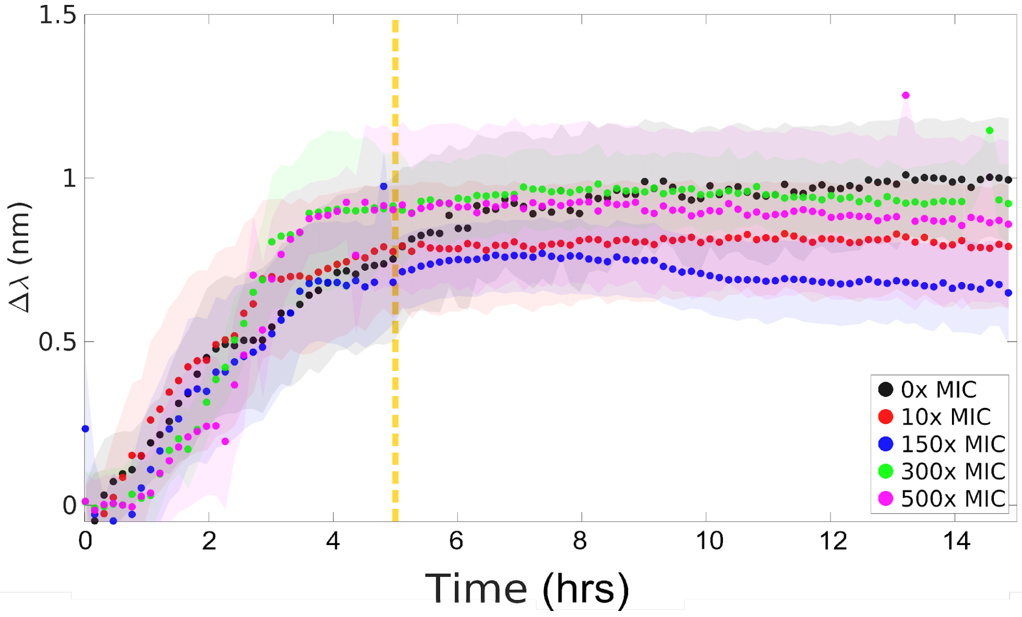

Supplementary Figure 5. Trimethoprim susceptibility testing in established biofilms. Trimethoprim at doses ranging from 10x to 500x MIC is introduced into the culture at 5 h (as indicated by the vertical dashed line). We observe no change within experimental accuracy, which suggests that trimethoprim has no effect on the biofilm.

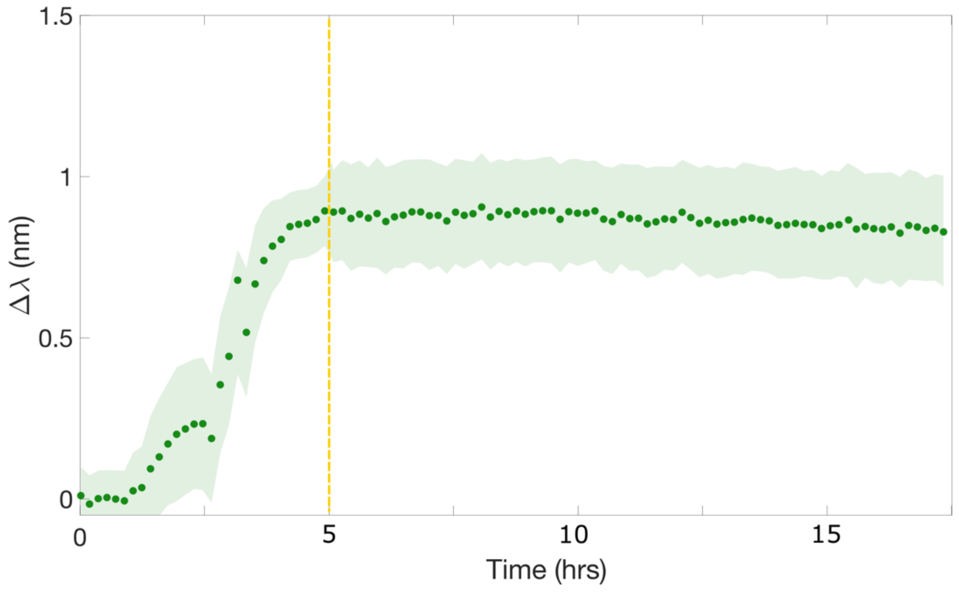

Supplementary Figure 6. Nitrofurantoin susceptibility testing in established biofilm. Nitrofurantoin with a concentration of 500x MIC is introduced into the culture at 5 h, and we observe no change in resonance wavelengths in the following 15 hours.

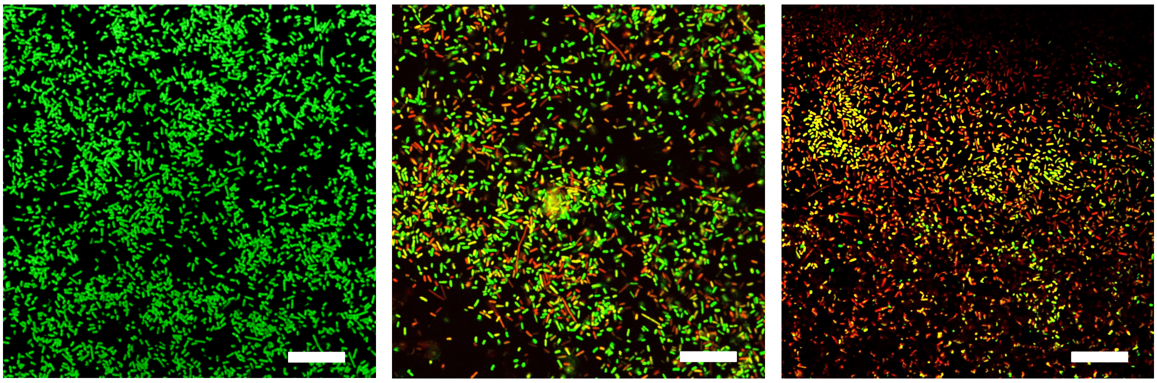

Supplementary Figure 7. Confocal microscopic images of biofilms on the grating surface after 10 h from the start of the experiments. With no antibiotic injected as a reference (left), with 150x MIC (middle), 500x MIC (right) of trimethoprim injected at 5 h from the start of the experiment. The biofilms were stained with the Live/Dead BacLight viability stain assay at 10 h and cells with compromised membranes stain red, whereas cells with intact membranes stain green. The scale bars in the images are 10 µm.

VII. Biofilm formation in bacteria spiked urine

Finally, we verify our technique's capability of working with a more complicated and biological growth culture, i.e. urine. A starting culture of *E.coli* TG1 was made from an overnight culture in LB broth. 50 mL cell suspension was spun down and washed three times in human urine (prefiltered using 0.2 µm pore size membrane filters). A suspension with *E.coli* inoculum density of 2 x 10^8 CFU per mL, was flown through the sensor with the resonance wavelength shift monitored, see Supplementary Figure 8. Interestingly, the sensor detected full biofilm coverage within 60 minutes, i.e. significantly faster than in LB. This demonstrates that our sensor is capable of operating with a relevant biological matrix.

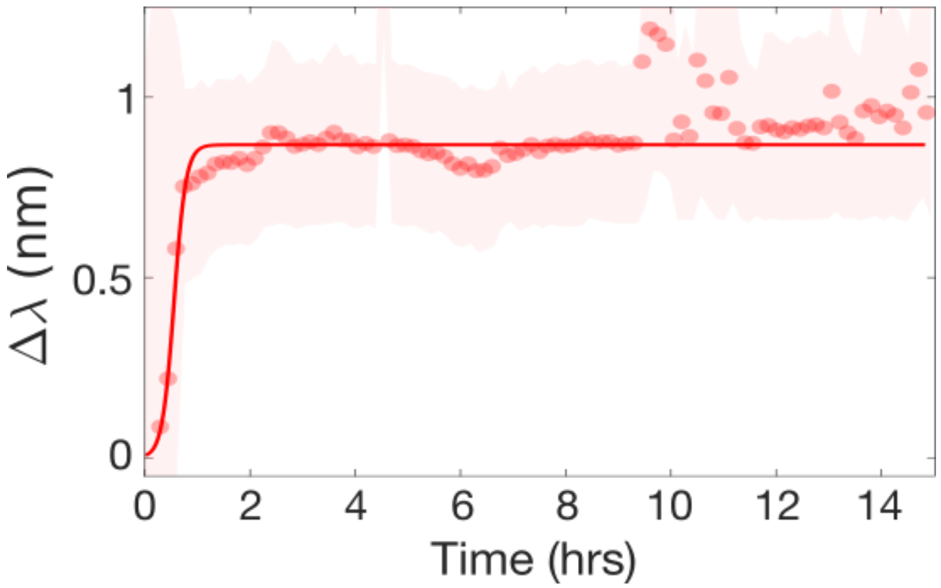

Supplementary Figure 8. Biofilm detection in bacterial spiked human urine. Biofilm growth is detected by the sensor in the flow cell with a suspension with *E.coli* inoculum concentration of 2 x 10^8 CFU per mL in human urine.
